# Genomics of glycopeptidolipid biosynthesis in *Mycobacterium abscessus *and *M. chelonae*

**DOI:** 10.1186/1471-2164-8-114

**Published:** 2007-05-09

**Authors:** Fabienne Ripoll, Caroline Deshayes, Sophie Pasek, Françoise Laval, Jean-Luc Beretti, Franck Biet, Jean-Loup Risler, Mamadou Daffé, Gilles Etienne, Jean-Louis Gaillard, Jean-Marc Reyrat

**Affiliations:** 1 Laboratoire de Microbiologie, Université de Versailles Saint-Quentin-en-Yvelines, Faculté de Médecine de Paris-Ile de France-Ouest, F-92380 Garches, France; 2 Inserm-UMR 570, Unité de Pathogénie des Infections Systémiques, Groupe Avenir, Paris Cedex 15, F-75730, France; 3 Université Paris Descartes, Faculté de Médecine René Descartes, Paris Cedex 15, F-75730, France; 4 Laboratoire Génome et Informatique, UMR CNRS 8116, F-91034 Evry cedex, France; 5 Institut de Pharmacologie et Biologie Structurale (UMR 5089), Département "Mécanismes Moléculaires des Infections Mycobactériennes", 205, route de Narbonne, F-31077 Toulouse-cedex, France; 6 INRA-Tours. UR918, F-37380 Nouzilly, France

## Abstract

**Background:**

The outermost layer of the bacterial surface is of crucial importance because it is in constant interaction with the host. Glycopeptidolipids (GPLs) are major surface glycolipids present on various mycobacterial species. In the fast-grower model organism *Mycobacterium smegmatis*, GPL biosynthesis involves approximately 30 genes all mapping to a single region of 65 kb.

**Results:**

We have recently sequenced the complete genomes of two fast-growers causing human infections, *Mycobacterium abscessus *(CIP 104536T) and *M. chelonae *(CIP 104535T). We show here that these two species contain genes corresponding to all those of the *M. smegmatis *"GPL locus", with extensive conservation of the predicted protein sequences consistent with the production of GPL molecules indistinguishable by biochemical analysis. However, the GPL locus appears to be split into several parts in *M. chelonae *and *M. abscessus*. One large cluster (19 genes) comprises all genes involved in the synthesis of the tripeptide-aminoalcohol moiety, the glycosylation of the lipopeptide and methylation/acetylation modifications. We provide evidence that a duplicated acetyltransferase (*atf1 *and *atf2*) in *M. abscessus *and *M. chelonae *has evolved through specialization, being able to transfer one acetyl at once in a sequential manner. There is a second smaller and distant (*M. chelonae*, 900 kb; *M. abscessus*, 3 Mb) cluster of six genes involved in the synthesis of the fatty acyl moiety and its attachment to the tripeptide-aminoalcohol moiety. The other genes are scattered throughout the genome, including two genes encoding putative regulatory proteins.

**Conclusion:**

Although these three species produce identical GPL molecules, the organization of GPL genes differ between them, thus constituting species-specific signatures. An hypothesis is that the compact organization of the GPL locus in *M. smegmatis *represents the ancestral form and that evolution has scattered various pieces throughout the genome in *M. abscessus *and *M. chelonae*.

## Background

*Mycobacterium abscessus *and *M. chelonae *are both species of rapidly growing mycobacteria (RGM) that have emerged as significant pathogens in humans during the last ten years: both species are major causes of skin and soft tissue infections following medical or surgical procedures [1]; *M. abscessus *also causes pulmonary infections and is increasingly recovered from patients with cystic fibrosis [1]. *M. chelonae *and *M. abscessus *are among the most-antibiotic resistant RGM species [1] and this has serious consequences for therapy [2].

**Figure 1:**
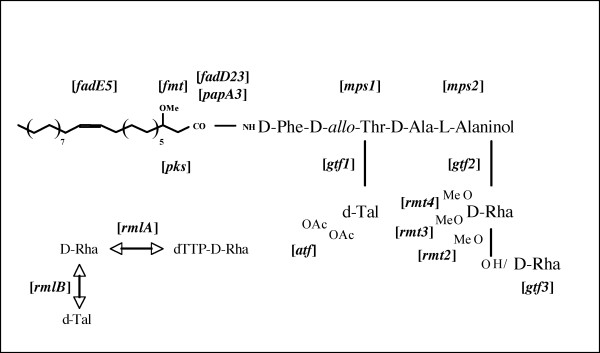
Schematic representation of the structure of the GPLs from *M. smegmatis*. The genes involved in the various metabolic steps are indicated in brackets. The locations of the variable substitutions linked to the common GPL core are shown. OAc: acetyl, OMe, methyl. dTal, 6-deoxytalose, D-Rha, rhamnose of the D series.

Glycopeptidolipids (GPLs) are surface lipids found on a number of mycobacterial species including both RGM (e.g., *M. smegmatis, M. chelonae, M. abscessus*) and slow growing mycobacteria (e.g., *M. avium *subsp. *avium*). These molecules can make up more than 70% of the lipids exposed at the bacterial surface (for a review see [3-5]). They have a glycosylated lipopeptide core that is variably modified by *O-*methylation and *O-*acetylation (Fig. 1). More precisely, the GPL structure is based on a tripeptide-aminoalcohol (D-Phe-D-*allo*-Thr-D-Ala-L-alaninol) *N-*linked to a long chain fatty acyl residue. This lipopeptide core is substituted by a *6*-deoxytalosyl (dTal) unit linked to the *allo*-Thr residue and by an *O*-methylated rhamnosyl unit linked to the terminal alaninol residue. The dTal residue can be *O-*acetylated on positions 3 and 4, further glycosylated on position 2 in *M. avium*, whereas the fatty acyl moiety and the rhamnosyl residue can be modified with one and three methyl groups on positions 2, 3, or 4, respectively and in some cases further glycosylated by the addition of an extra rhamnosyl unit [3,11,6] (Fig. 1).

GPLs are required for sliding motility, biofilm formation and for maintaining cell wall integrity [7,8]. They also influence bacterial aggregation [7,9], induce the release of prostaglandin E2 and interfere with the interaction between mycobacteria and human monocytes/macrophages [10,11]. Moreover, several recent studies show that natural variants of *M. abscessus*, which produce only small amounts of GPL are more invasive than the high-level producers [12-14]. Thus, GPLs appear to play an important role in both the physiology and the pathogenicity of mycobacteria.

The complete genomes of *M. chelonae *(CIP 104535T) and *M. abscessus *(CIP 104536T) have recently been sequenced to help to elucidate their molecular mechanisms of pathogenicity and antibiotic resistance. By exploiting available data concerning the genetic basis of the GPL biosynthetic pathway in the RGM model organism, *M. smegmatis*, we identified and analysed the genetic regions encoding enzymes involved in GPL biosynthesis in *M. chelonae *and *M. abscessus*.

## Results

### Biochemical analysis of the glycopeptidolipid produced by *M. abscessus *and *M. chelonae*

**Figure 2:**
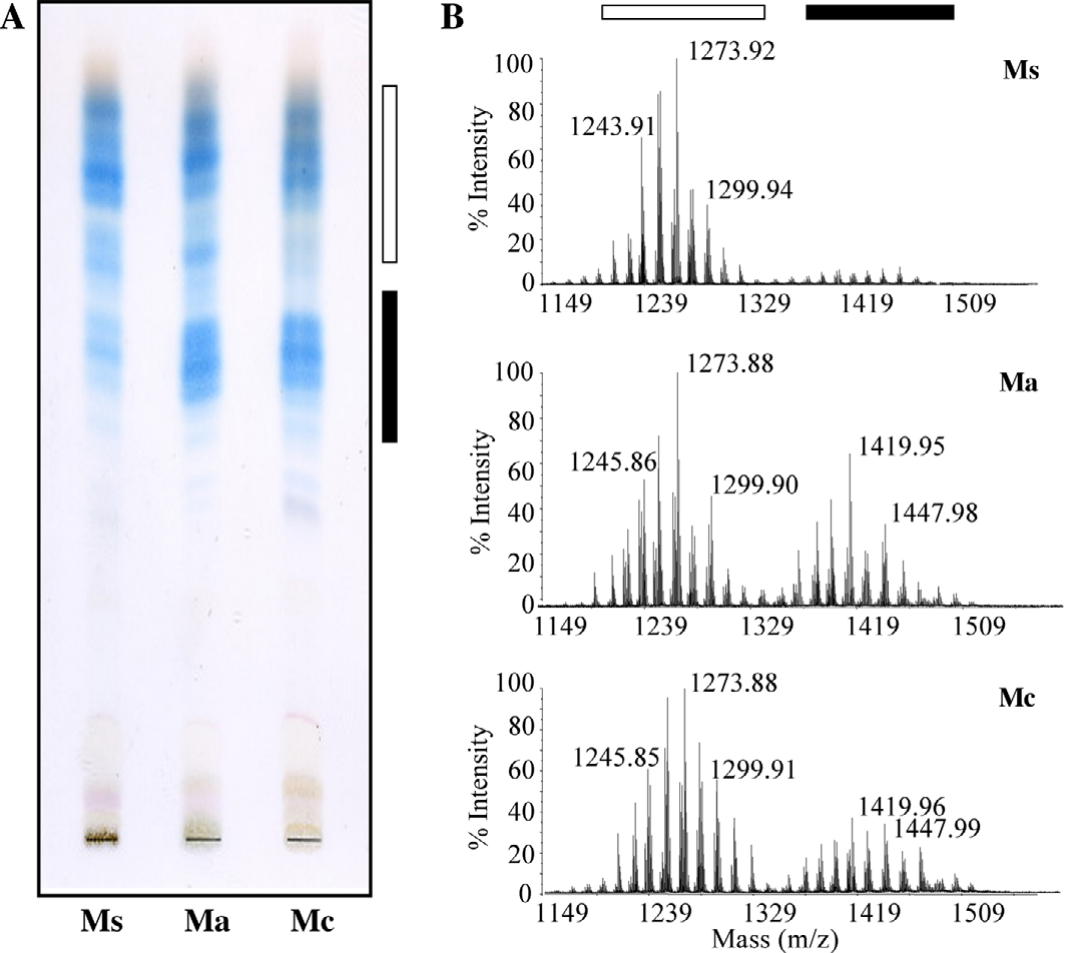
**A) **Thin-layer chromatography analysis of the crude lipid extract of *M. smegmatis *(Ms), *M. abscessus *(Ma), and *M. chelonae *(Mc). Open rectangle, diglycosylated GPLs; closed rectangle, triglycosylated GPLs. **B) **MALDI-TOF mass spectra of the crude lipid fractions of *M. smegmatis *(Ms), *M. abscessus *(Ma), and *M. chelonae *(Mc). Open rectangle, diglycosylated GPLs; closed rectangle, triglycosylated GPLs.

Since GPL molecules may differ at a structural level, we first assessed the GPL status of the strains used in this study for comparative genomic analysis: *M. smegmatis *mc^2^155, *M. chelonae *CIP 104535T and *M. abscessus *CIP 104536T. Strains were grown in both early exponential and late stationary phases (Fig. 2A) and lipids were extracted from cells [11]. The GPLs were identified by thin-layer chromatography (TLC), a method separating molecules according to their hydrophobic index, and using matrix-assisted laser desorption/ionization time of flight (MALDI-TOF) mass spectrometry analysis [11]. Since GPLs contain sugar moieties, GPL-like compounds were identified by anthrone staining [7]. Whatever the growth phase, the three strains produced similar diglycosylated GPLs, and in comparable quantities; however, triglycosylated GPLs were clearly more abundant in *M. chelonae *and *M. abscessus *than in *M. smegmatis *in both conditions (Fig. 2A). The precise nature of the GPL-like compounds was resolved by MALDI-TOF mass spectrometry, a very accurate and sensitive method for detecting glycolipids. The molecular masses of all the GPL-associated pseudomolecular ions peaks detected in the mass spectra of all strains perfectly matched the previously calculated molecular weights of the GPLs in these strains. Most of the GPLs produced by *M. smegmatis *mc^2^155 were diglycosylated forms, confirming earlier reports [15]. *M. abscessus *and *M. chelonae *produced the same diglycosylated GPLs ([M+K+] m/z at 1273 amu), and in addition produced a higher amount of triglycosylated GPLs than *M. smegmatis *([M+K+] m/z at 1419 amu) as expected from TLC analysis (Fig. 2B).

### Comparative genomics of the GPL biosynthetic pathway

**Figure 3:**
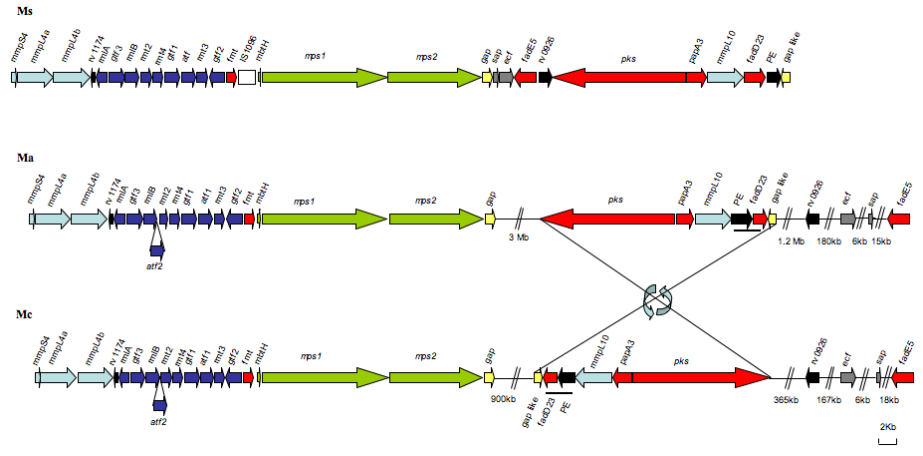
Genetic organization of the GPL locus in *M. smegmatis *(Ms), *M. abscessus *(Ma), and *M. chelonae *(Mc). The ORFs are depicted as arrows and have been drawn to scale. Colour code: Light blue: *mmpL *family, black: unknown, purple: sugar biosynthesis, activation, transfer and modifications, red: lipid biosynthesis, activation, transfer and modifications, green: pseudopeptide biosynthesis, yellow: required for GPL transport to the surface, grey: regulation.

Comparative genomic analysis was performed by pairwise alignments of the proteins of the GPL locus of *M. smegmatis *and each of the complete proteomes of *M. abscessus *and *M. chelonae *[16,17]. All the genes necessary for GPL biosynthesis are clustered in a single region of the *M. smegmatis *genome, thus constituting a true "GPL locus" (Figure 3). In this species, most of the genes of the locus (Fig. 1) have been identified experimentally and/or by *in silico *prediction (Table 1). The tripeptide-aminoalcohol moiety is non-ribosomally assembled by the product of the *mps1 *and the *mps2 *genes [18,19], and the lipid synthesis and attachment to the tripeptide-aminoalcohol moiety probably require the concerted action of several gene products (*pks*, *fadD23*, *papA3*) [19,20]. The genes involved in the glycosylation of the lipopeptide core (*gtf1*, *gtf2*) were recently characterized by selectively inactivating them both and by biochemical analysis of the resulting mutants [21]. Triglycosylated GPLs, described as being produced mostly during stationary phase [22], result from the addition of an extra rhamnosyl residue (*gtf3*) [9,21]. The genes involved in the *O-*methylation of the various hydroxyl groups of the rhamnosyl unit (*rmt2*, *rmt3 *and *rmt4*) and of the lipid moiety (*fmt*) of the GPLs have been characterized using similar methods [23-25]. The *atf *gene product is responsible for acetylating two particular hydroxyl residues of the dTal [26], and FadE5 probably introduces the double bond into the fatty acid. The *rmlA *and *rmlB *genes are associated with the activation and epimerisation of the sugars [27]. Two members of the *mmpL *family, a group of genes encoding large membrane proteins, are also required for GPL biosynthesis [19,26]. The exact function of these proteins, MmpL4a and MmpL4b, is not known in detail although a recent study performed in *M. tuberculosis *suggests that MmpL proteins (MmpL7) may channel the polyketide products during their synthesis by the polyketide synthase, coupling synthesis and export [28]. Finally, the transport of the GPLs to the surface of the bacilli involves the integral membrane protein Gap [19]. This locus also contains eight other genes that have not yet been experimentally characterized: two encode membrane proteins (*mmpS4 *and *mmpL10*) that may also interact with the polyketide synthase of the cluster, two encode a sigma factor and a sigma-associated protein (*ecf *and* sap*, respectively) that are believed to contribute to the regulation of GPL production, and four have no known function (Table 1 and Fig. 1) [18,19].

All the genes of the *M. smegmatis *GPL locus have close orthologs in both *M. chelonae *and *M. abscessus *(Table 1). These orthologs share more than 80% of identity with each other and most are more than 90% identical (data not shown). The percentage of identity between *M. chelonae/M. abscessus *and *M. smegmatis *orthologs ranges between 30 and 89%, with two-thirds of *M. chelonae/M. abscessus *orthologs being ≥70% identical to their *M. smegmatis *counterparts. Identity is less than 50% for only four orthologs: *sap*, *ecf*, *Rv0926 *and *Rv1174c*. The functions of *Rv0926 *and *Rv1174c *are not known, *sap *and *ecf *may play roles in the regulation of GPL biosynthesis, and were this the case, it would suggest that the regulatory circuits in these species have diverged.

Unlike *M. smegmatis*, the *M. chelonae/M. abscessus *GPL orthologs are not gathered in a single region (Figure 3). In both *M. chelonae *and *M. abscessus*, there is a large region containing 19 genes (*mmpS4 *to *gap*). This region contains all genes involved in the synthesis of the tripeptide-aminoalcohol moiety, the glycosylation of the lipopeptide and the *O-*methylation and *O-*acetylation modifications (see also Fig. 1). This region is very similar to the corresponding region of the *M. smegmatis *GPL locus, except for the two following differences. First, there is no mobile element, either upstream of *mbtH *like in *M. smegmatis *mc^2^155 or at any other location. Second, there are two *atf *orthologs (we called them *atf1 *and *atf2*) in both *M. chelonae *and *M. abscessus*: *atf1 *is at the same location as *atf *in *M. smegmatis *whereas *atf2 *is inserted between *rmlB *and *rmt2*. *M. chelonae atf1 *and *atf 2 *genes are 58% identical (71% similarity), and are 76 and 60% identical to *M. smegmatis atf *respectively (88 and 75% similarity respectively); *M. abscessus atf1 *and *atf 2 *genes are 57% identical (72% similarity), and are 72 and 59% identical to *atf *respectively (83 and 74% similarity respectively). There is a smaller region forming a block of 6 genes 900 kb from this first region in *M. chelonae *and 3 Mb away in *M. abscessus*. These six genes (*pks *to *gap*-like) are probably involved in the lipid synthesis and attachment to the tripeptide-aminoalcohol moiety (e.g., *pks*, *fadD23*, *papA3*), but *pks *is the only one that has been experimentally studied so far [19]. This block is part of a large region that is inverted between *M. chelonae *and *M. abscessus*. It is very similar to the corresponding part of the *M. smegmatis *GPL locus except that the order of the *pe *and the *fadD23 *genes is switched in *M. chelonae/M. abscessus *relative to *M. smegmatis*. Finally, four genes closely linked in *M. smegmatis *(*Rv0926*, *fadE5*, *sap*, *ecf*) are scattered on the chromosome in *M. chelonae *and *M. abscessus*, with distances differing between species.

To test whether the locus organization was a particularity of the sequenced strains (CIP 104535T and CIP 104536T), 5 clinical isolates of each species were analyzed by PCR using two couples of primers (Additional file 1). All the *M. abscessus *and *M. chelonae *isolates had the same PCR pattern. This experiment shows that the genetic organization of the GPL locus depicted in Figure 3 is not strain-dependant but is probably valid for the whole species.

### Acetyltransferases have evolved specificity in *M. abscessus *and *M. chelonae*

As seen above, there is only one *atf *in *M. smegmatis *while there are two in both *M. abscessus *and *M. chelonae*. *In M. smegmatis*, Atf catalyses the transfer of two acetyl groups onto the dTal moiety (on positions 3 and 4) [26]. We consequently tested whether the presence of the 2 *atf *in the other species was a redundant or specialization process and used *atf1 *and *atf2 *of *M. abscessus *as a model. We used a *M. smegmatis atf- *mutant as a recipient host and complemented it with constructs expressing the various *atf *genes (Additional file 2). The GPLs produced by the various complemented strains were analysed by both TLC and MALDI-TOF. The GPLs produced by the *M. smegmatis atf- *mutant were, as expected, non *O-*acetylated ([M+K+] m/z at 1189 and 1215 amu) as deduced from the value of the masses of the pseudomolecular ions and its altered migration on TLC. Reintroduction of the *atf *gene of *M. smegmatis *led to the production of *O-*diacetylated GPL ([M+K+] m/z at 1273 and 1299 amu) with a wild type migration pattern. Complementation by the *atf1 *gene of *M. abscessus *was enabling the production of mono-*O-*acetylated GPL ([M+K+] m/z at 1231 and 1258 amu) having an intermediary migration between the di-*O-*acetylated and the non-acetylated forms, indicating a specialisation process. Surprisingly, the GPL produced by the *M. smegmatis atf- *mutant complementated by the *atf2 *gene of *M. abscessus *was most exclusively non-acetylated as judged by TLC (Additional file 2) and MALDI-TOF analysis confirmed molecular masses of 1189 and 1215 amu (data not shown). However, when both *atf1 *and *atf2 *were simultaneously introduced into the host strain, the production of di-*O-*acetylated GPLs was restored as judged by TLC (Additional file 2) and MALDI-TOF analysis confirmed molecular masses of 1273 and 1299 amu (data not shown). This set of experiments shows that the *atf2 *gene of *M. abscessus *is fully functional and needs a mono-*O-*acetylated dTal substrate to be able to transfer the second acetyl moiety. In conclusion, the acetyltransferases encoded by the *M. abscessus *GPL locus are not redundant but have evolved specificity, being able to transfer one acetyl at once in a sequential manner.

## Discussion

This study is the first addressing the genetics of GPL biosynthesis in two clinically significant RGM species, *M. chelonae *and *M. abscessus*. The major observation is that, despite producing structurally identical GPL molecules, the genes necessary for its biosynthesis are organized very differently. In *M. smegmatis*, the GPL locus is made up of almost 30 genes in a region of ~65 kb, and therefore does not comply with the prokaryotic rule of 1 gene/kb. This is because GPL biosynthesis involves very large multi-modular proteins, for example the non-ribosomal protein synthetases (Mps1 and Mps2) and the polyketide synthase (Pks), and consequently very long genes. Several genes appear to be organized into operons, one of which has been identified formally and contains *mbtH*, *mps1, mps2, gap*, *sap *and *ecf *[19]. Interestingly, a mobile element, IS*1096*, is located just upstream from *mbtH *in mc^2^155 strain. This upstream region corresponds to the promoter of the *mbtH *operon and may therefore interfere with the expression of this operon, as it does in other biological systems [29,30]. Surprisingly, *M. chelonae *and *M. abscessus *produced clearly more triglycosylated GPL than *M. smegmatis*. This observation argues in favour of differences in *gtf3 *expression in these three species.

All the genes are clustered in *M. smegmatis*, but are scattered in several blocks in *M. chelonae *and *M. abscessus*. The various genomic pieces correspond to blocks of function: one block corresponds to the synthesis of the tripeptide-aminoalcohol moiety, the glycosylation of the lipopeptide and *O-*methylation/acetylation modifications, and another to lipid biosynthesis and its attachment to the tripeptide-aminoalcohol moiety. In addition, these species differ by one inversion and one duplication. An attractive hypothesis is that the compact organization of the GPL locus in *M. smegmatis *represents the ancestral form and that evolution has scattered various pieces throughout the genome in *M. abscessus *and *M. chelonae*. However, the opposite hypothesis in which genes involved in a metabolic pathway would have the tendency to gather during evolution cannot be excluded. The fact that both *M. chelonae *and *M. abscessus *have two non-redundant *O-*acetyltransferases suggests that *atf2 *may have arisen from the duplication of *atf1*. Interestingly, *atf1 *is less similar to *atf2 *than to *M. smegmatis atf*, also indicating a functional divergence between *atf1 *and *atf2*. In *M. smegmatis*, *atf *mediates the *O-*acetylation of the dTal in both positions 3 and 4. We showed that, in *M. abscessus*, *atf1 *and *atf2 *are each specifically responsible for one of these two reactions and that probably act sequentially.

We showed that the GPL biosynthetic pathway is highly conserved between *M. chelonae *and *M. abscessus*, consistent with the close relatedness of these two species [31]. However, due to genomic rearrangements between the two species, the two blocks are located at different coordinates and the block of six genes is inverted in these two species with respect to that in *M. smegmatis*. These genomic rearrangements are consistent with the separation of the two species that were formerly parts of a single complex [31]. We showed, using a panel of clinical isolates that these differences are species-specific, and may thus be used as a discriminative marker. The genomic findings are in agreement with the biochemical data showing that the two species produce structurally identical GPL molecules [3]. However, differences in terms of regulation cannot be excluded and it is not known whether additional genes are needed for GPL biosynthesis, export and regulation in these three species.

*M. chelonae *and *M. abscessus*, like other mycobacterial species [32,33], can naturally switch from a rough (R) to smooth (S) and from a S to a R morphotype [12,13]. R strains are associated with a low GPL production, high invasive ability and a higher virulence in the mouse model [13,14]. However, despite several attempts, the genetic bases for this natural S/R switching remain obscure. Several studies using *M. avium *and *M. smegmatis *describe various genes involved in the transition between S and R morphotypes, most of which are implicated in the GPL biosynthetic pathway [18,19,34]. The identification of the genes required for the synthesis and export of these metabolites should help our understanding of the natural variation in the morphology and virulence variation of these species.

## Conclusion

We showed that *M. abscessus *and *M. chelonae *contain genes corresponding to all those of the *M. smegmatis *"GPL locus" with an extensive conservation of the predicted protein sequences. This finding is consistent with the production of GPL molecules indistinguishable by either thin-layer chromatography or mass spectrometry. Despite, the genomic and structural homology, the GPL locus appears to be split into several parts in *M. chelonae *and *M. abscessus*. One large cluster (19 genes) comprises all genes involved in the synthesis of the tripeptide-aminoalcohol moiety, the glycosylation of the lipopeptide and *O-*methylation/acetylation modifications. A second smaller and distant (*M. chelonae*, 900 kb; *M. abscessus*, 3 Mb) cluster of six genes is involved in the synthesis of the fatty acyl moiety and its attachment to the tripeptide-aminoalcohol moiety. The other genes are scattered throughout the genome, including two genes encoding putative regulatory proteins. Although these three species produce identical GPL molecules, the organization of GPL genes differs between them, thus constituting species-specific signatures. An attractive hypothesis is that the compact organization of the GPL locus in *M. smegmatis *represents the ancestral form and that evolution has scattered various pieces throughout the genome in *M. abscessus *and *M. chelonae*, although the opposite scenario cannot be excluded.

## Methods

### Bacterial strains

*M. smegmatis *mc^2^155 and *M. abscessus *CIP104536T (ATCC 19977T) were cultured in 7H9 supplemented with 10% ADC at 37°C. *M. chelonae *CIP 104535T (ATCC 35752T) was cultured in the same medium at 30°C. All bacterial cultures were harvested in either early exponential or late stationary phase. When required, antibiotics were included at the following concentrations: kanamycin, 50 μg/ml, hygromycin, 200 μg/ml (for *E. coli*) or 50 μg/ml (for mycobacteria).

### Lipid analysis

Lipids were extracted from cells with a mixture of chloroform and methanol and further partitioned by methanol precipitation as previously described [11]. The GPLs (250 μg lipid each deposit) were identified by TLC on silica gel Durasil 25-precoated plates (Macherey-Nagel) run in chloroform/methanol (90:10 [vol/vol]) and using MALDI-TOF mass spectrometry analysis [11]. These sugar-containing compounds were identified by spraying plates with 0.2% anthrone in concentrated sulfuric acid, followed by heating at 110°C [7].

### Computer methods

The accession number of the GPL locus of *M. smegmatis *is AY439015. The sequencing and the assembly of the genome of *M. abscessus *and *M. chelonae *was performed by the CNS (Centre National de Séquençage-Evry-France), [35]. Open reading frames of both *M. abscessus *and *M. chelonae *were predicted using both SHOW [36] and ARTEMIS [37]. The accession numbers corresponding to the regions of the GPL locus of *M. abscessus *are AM31616 to AM31621. The accession numbers corresponding to the regions of the GPL locus of *M. chelonae *are AM231610 to AM231615. The complete sequence of *M. abscessus *and *M. chelonae *will be reported elsewhere (J. L Risler & J. L Gaillard, unpublished data). The 6901 putative proteins of the genome of *M. smegmatis *were obtained from The Institute for Genomic Research [38]. The comparative genomic analysis was performed by pairwise alignments between the proteins of the GPL locus of *M. smegmatis *and each of the complete proteomes mentioned above. These comparisons were performed using the LASSAP software and Z-values were calculated as described [16,17]. The identification of the orthologous links was performed using the results of the pairwise comparisons as follows: For each gene of the GPL locus of *M. smegmatis*, 5 bi-directional best hits (BBH) were identified. The BBH having the best Z-value was selected. When several BBH exhibited a similar Z-value (some of the genes of the GPL locus such a *fadD *and *fadE *are affected by a high degree of paralogy), the gene preserving the syntenic context was selected. Identity below 25% was not considered as significant. All the selected orthologs have a Z-value greater than 14 (except the *sap *gene and its orthologs).

### Analysis of the *M. abscessus *and *M. chelonae *clinical isolates using PCR

The chromosomal DNA was prepared using the bead-beater-phenol extraction method. The bacterial pellet (corresponding to 50 ml culture) were suspended in 5 ml of solution I (25% sucrose; 50 mM TrisCl 1 M pH = 8; 50 mM thiourea; 10 mg/ml lysozyme). The thiourea inhibits the Tris-dependent DNAse that is present in some strains [39]. Solution II (25% sucrose; 50 mM TrisCl pH = 8; 50 mM EDTA pH = 8) was added and the bacterial cells were lysed as described by Howard and *al. *[40]. Proteinase K was added to the lysate at 100 μg/ml and incubated over-night at 55°C. The DNA was extracted using phenol/chlorophorm/isoamyl-alcohol (25:24:1) and precipitated with propanol. Primers (mpsF1, mpsF2, mpsR; pkF1, pkF2, pkR) (Additional file 3) were designed according to the chromosomal sequence of *M. abscessus *and *M. chelonae*. PCR amplification was performed using Dynazyme Taq polymerase according to manufacturer instructions (Finnzyme, Espoo, Finland).

#### Construction of acetyltransferase expression plasmids

The wild type *M. smegmatis atf *gene (accession number AY138899) coding sequence was amplified by PCR using the Pfu DNA Polymerase (Stratagene), the genomic DNA of *M. smegmatis *mc^2^155 as template and primers containing an engineered *Xba*I site (atfsmeg.5 and atfsmeg.3) (Additional file 3). After purification with the PCR purification Qiagen kit, PCR products were digested with *Xba*I and cloned into the dephosphorylated expression vector pNIP40b [41] at the unique *Xba*I site to generate pNIP*atfsmeg*. One clone having the *atfsmeg *gene inserted in the opposite direction of the hygromycin resistant gene was selected and sequenced. Using *M. abscessus *ATCC 19977^T ^genomic DNA as template, a similar strategy was applied to clone *atf1 *gene and *atf2 *gene (AM231618) using primers atf1abs.5/atf1abs.3 and primers atf2abs.5/atf2abs.3 (Additional file 3) into pNIP40b [41] yielding pNIP*atf1absc *and pNIP*atf2absc*, respectively. To clone the *M. abscessus atf1 *and *atf2 *genes in frame, the *af1absc *PCR product was digested by *Cla*I and the *atf2 *gene was amplified using new primers (atf2ClaI.5 and atf2abs.3) and digested with *Cla*I. The 2 PCR products were digested with *Xba*I, purified and ligated, with the dephosphorylated expression vector pNIP40b at its unique *Xba*I site to generate pNIP*atf1_2absc*. These plasmids were electroporated into *M. smegmatis *mc^2^155 *atf- *mutant [26] and transformants were selected on plates containing kanamycin and hygromycin. These strains are named *atf-/atfsMs*, *atf-/atf1Ma*, *atf-/atf2Ma *and *atf-/atf1_2Ma*.

## Abbreviations

GPL: glycopeptidolipid

RGM: rapidly growing mycobacteria

dTal: 6-deoxytalose

D-Rha: rhamnose of the D series

TLC: thin-layer chromatography

MALDI-TOF: matrix-assisted laser desorption/ionization time-of-flight

amu: atomic mass unit

## Authors' contributions

FR carried out the bioinformatic studies, analysed the Ma and Mc strains by PCR and drafted the manuscript. CD carried out the molecular biology experiments, participated in the sequence alignment and drafted the manuscript. SP participated in the sequence alignment. FL and JLB produced and analysed the MS data. FB collected and cultivated various *M. avium *subsp. *avium* strains used as internal control and helped to draft the manuscript. JLR participated in the sequence alignments and in the phylogenetic analysis. MD participated in the analysis of the biochemical experiments. JLG participated in the design of the study and helped to draft the manuscript. GE performed biochemical experiments, participated in the design of the study and helped to draft the manuscript. JMR conceived of the study, participated in its design and coordination and drafted the manuscript. All authors read and approved the final manuscript.

## Supplementary Material

Additional File 1 Analysis of various clinical isolates of *M. abscessus *and *M. chelonae *by PCR. Click here for file

Additional File 2 **A) **Thin-layer chromatography analysis of the crude lipid extracts of wild-type *M. smegmatis *(1), the *atf- *mutant (2), the *atf- *mutant complemented by the *atf *gene of *M. smegmatis *(3), the *atf1 *(4) or *atf2 *(5) genes of *M. abscessus or *both (6). **B) **MALDI-TOF mass spectra of the crude lipid fractions of the various *M. smegmatis atf *complemented strains. Click here for file

Additional File 3 List of the oligonucleotides used in this study. Click here for file

## Figures and Tables

**Table 1:** Genes of the *M. smegmatis *GPL locus and their orthologs in *M. abscessus *and *M. chelonae*.

*M. smegmatis*			*M. abscessus*		*M. chelonae*	
Gene	Gene n°	Proposed function^(a)^	Gene n°	%^(b)^	Gene n°	%^(b)^

*mmpS4*	MSMEG0373	Membrane associated. Interaction with Pks, °	ma4117c	78	mc1618	74
*mmpL4a*	MSMEG0374	Membrane associated. Interaction with Pks, °	ma 4116c	78	mc1619	77
*mmpL4b*	MSMEG0375	Membrane associated. Interaction with Pks, °	ma4115c	76	mc1620	75
*Rv1174*	MSMEG0376	None	ma4114	48	mc1621c	48
*rmlA*	MSMEG0377	Alpha-D-hexose-1-phosphate-thymidylyl-transferase (EC 2.7.7.24), °	ma4113	85	mc1622c	85
*gtf3*	MSMEG0378	D-Rhamnose rhamnosyltransferase, *+*	ma4112c	49	mc1623	71
*rmlB*	MSMEG0379	UDP-hexose 4-epimerase, °	ma4111c	77	mc1624	78
*rmt2*	MSMEG0380	Rhamnose 2-O-methyltransferase, *+*	ma4109c	72	mc1626	70
*rmt4*	MSMEG0381	Rhamnose 4-O-methyltransferase, *+*	ma4108c	83	mc1627	83
*gtf1*	MSMEG0382	D-*allo*-threonine 6-deoxytalosyltransferase, *+*	ma4107c	76	mc1628	77
*atf*	MSMEG0383	Integral membrane protein. 6-deoxytalose 3,4-O-acetyltransferase, +	ma4106c^(c)^	72	mc1629^(c)^	76
			ma4110c^(d)^	59	mc1625^(d)^	60
*rmt3*	MSMEG0384	Rhamnose 3-O-methyltransferase, *+*	ma4105c	81	mc1630	82
*gtf*	MSMEG0385	L-alaninol rhamnosyltransferase, *+*	ma4104	67	mc1631c	72
*fmt*	MSMEG0386	Fatty acid O-methyltransferase, *+*	ma4103c	67	mc1632	62
*mbtH*	MSMEG0387	None	ma4100c	89	mc1635	89
*mps1*	MSMEG0390	Non-ribosomal protein synthase. Synthesis of the dipeptide, +°	ma4099c	70	mc1636	70
*mps2*	MSMEG0392	Non-ribosomal protein synthase. Synthesis of the amino acid alcohol, +°	ma4098c	71	mc1637	72
*gap*	MSMEG0393	Integral membrane protein. Required for GPL export, *+*	ma4097c	58	mc1638	55
*sap*	MSMEG0394	Sigma-associated protein	ma4454c	30	mc1299	30
*ecf*	MSMEG0395	Sigma factor of the ECF family. Required for regulation	ma4459c	46	mc1294	47
*fadE5*	MSMEG0396	Fatty acid desaturase, °	ma4437	78	mc1318c	78
*Rv0926*	MSMEG0397	None	ma4633	36	mc1136c	36
*pks*	MSMEG0398	Fatty acid synthesis and activation, +°	ma0939	79	mc0819	79
*papA3*	MSMEG0399	Transfer of the Pks-bound fatty acid to the pseudotetrapeptide, °	ma0938c	77	mc0818c	77
*mmpL10*	MSMEG0400	Membrane associated. Interaction with Pks, °	ma0937c	75	mc0817c	74
*fadD23*	MSMEG0401	Long chain fatty acyl-AMP ligase, °	ma0935c	73	mc0815c	74
*pe*	MSMEG0402	None	ma0936c	64	mc0816c	66
*gap-like*	MSMEG0403	Integral membrane protein. Role in the transport of GPLs, °	ma0934	55	mc0814	55

^(a) ^+, experimentally validated function; °, *in silico *predicted function.

^(b) ^Percentage of identity between *M. abscessus *or *M. chelonae *genes and *M. smegmatis *genes.

^(c) ^*atf1 *(see text and legend to Fig. 3).

^(d) ^*atf2 *(see text and legend to Fig. 3).
