# Supplementary figures and images for: Genomics of glycopeptidolipid biosynthesis in Mycobacterium abscessus and M. chelonae
Source: BMC Genomics. 2007 May 9;8:114. doi: 10.1186/1471-2164-8-114 (PMC1885439; doi:10.1186/1471-2164-8-114)

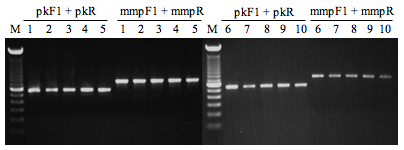

Supplement: Additional File 1 — Analysis of various clinical isolates of M. abscessus and M. chelonae by PCR. [file 1471-2164-8-114-S1.tiff]

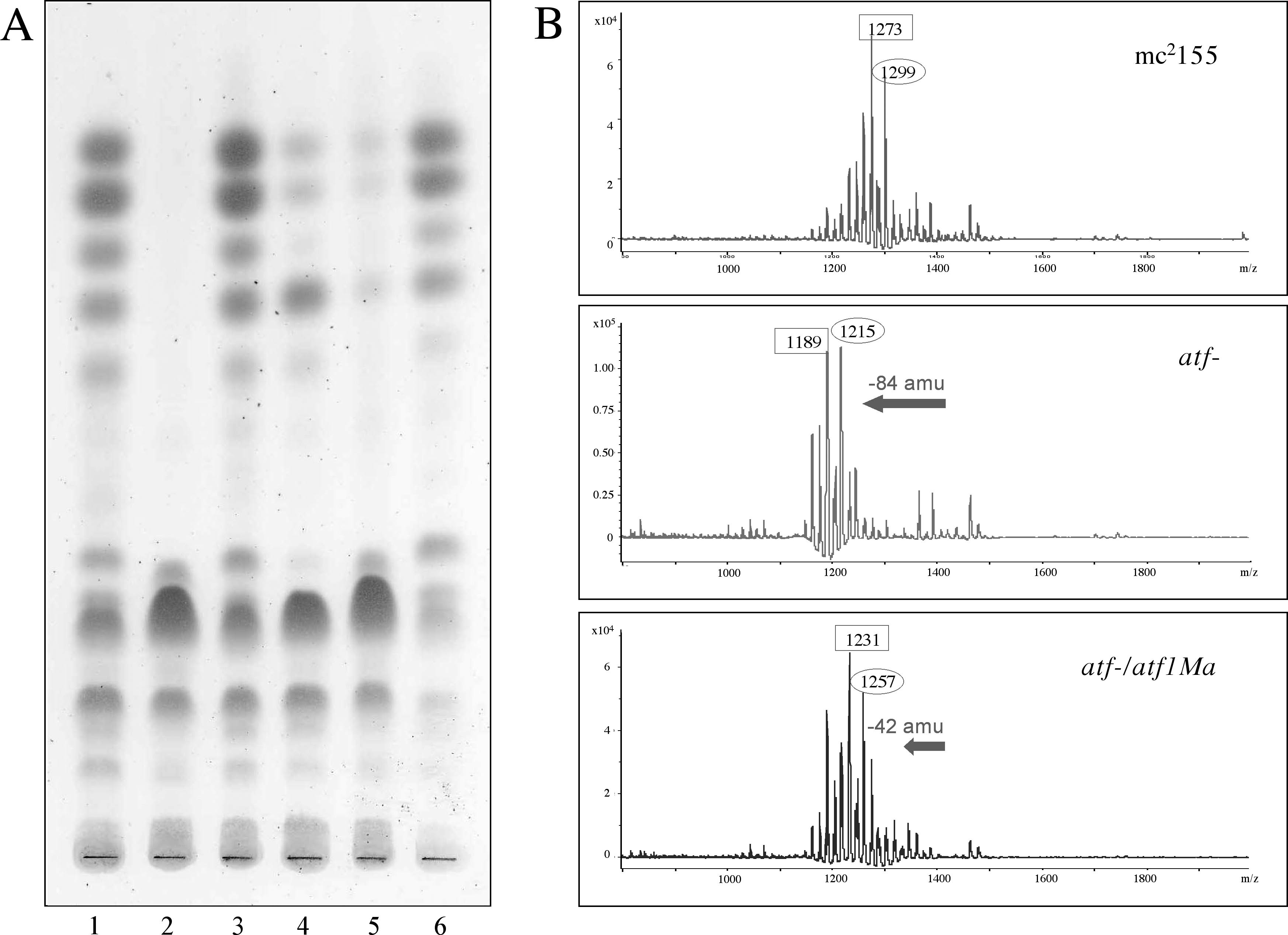

Supplement: Additional File 2 — A) Thin-layer chromatography analysis of the crude lipid extracts of wild-type M. smegmatis (1), the atf- mutant (2), the atf- mutant complemented by the atf gene of M. smegmatis (3), the atf1 (4) or atf2 (5) genes of M. abscessus or both (6). B) MALDI-TOF mass spectra of the crude lipid fractions of the various M. smegmatis atf complemented strains. [file 1471-2164-8-114-S2.tiff]
