# Supplementary material for: Genomics of glycopeptidolipid biosynthesis in Mycobacterium abscessus and M. chelonae
Source: BMC Genomics. 2007 May 9;8:114. doi: 10.1186/1471-2164-8-114 (PMC1885439; doi:10.1186/1471-2164-8-114)
Supplement: Additional File 3 — List of the oligonucleotides used in this study. [file 1471-2164-8-114-S3.doc]

**Supplementary Table 1.** List of primers used in this study.

| Primers | Sequences (5’-3’) |
| --- | --- |
| atfsmeg.5 | ctag**tctaga**caagctctcgcacgaggaatgacc |
| atfsmeg.3 | ctag**tctaga**agcgcggaccgaaagtccatttgc |
| atf1abs.5 | ctag**tctaga**aatcctggcgtcggactatgccga |
| atf1abs.3 | ctag**tctaga**gaagtgtgcgcgcaagccaatact |
| atf2abs.5 | ctag**tctaga**gatcgacaaagactgcccgaatcg |
| atf2abs.3 | ctag**tctaga**cggcccgagggcatcgagcatacga |
| atf2ClaI.5 | gcta**atcgat**ccatgaagctcggttctgta |
| mpsF1 | gagtcgcgacaccgtaaatcc |
| mpsF2 | ggaatccacagacgcatcaaaa |
| mpsR | cgagcgcggcctgactgaacc |
| pkF1 | gggtcccgcggcagcatct |
| pkF2 | ccagttcggcggcaagttcacg |
| pkR | gcgggtgggcgagttgac |

Engineered *Xba*I and *Cla*I sites are printed in bold.
